# Intérêt de l'inspection visuelle à l'acide acétique et au soluté de Lugol avec colposcope dans le dépistage des lésions du col utérin au Gabon

**DOI:** 10.11604/pamj.2015.22.165.7038

**Published:** 2015-10-21

**Authors:** Édith Mpiga, Mahinè Ivanga, Ismaël Hervé Koumakpayi, Corinne Engohan-Aloghe, Junie Chansi Ankély, Ernest Belembaogo, Jean-François Meye

**Affiliations:** 1 Hôpital d'Instruction des Armées Omar Bongo Ondimba, Libreville, Gabon; 2 Centre Hospitalier Universitaire d'Angondjé, Service de Gynécologie Obstétrique, Libreville, Gabon; 3 Institut de Cancérologie de Libreville, Département de Biologie des tumeurs et d'Anatomie Pathologique, Libreville, Gabon; 4 Institut de Cancérologie de Libreville, Département d'Oncologie Médicale, Libreville, Gabon

**Keywords:** Col de l′utérus, lésions précancéreuses, cancer, IVA/IVL, FCV, Gabon, Cervix, precancerous lesions, cancer, VILI, Pap smear, Gabon

## Abstract

**Introduction:**

Au Gabon, le dépistage des lésions précancéreuses et cancéreuses du col de l'utérus n'est pas systématique. La vulgarisation de ce dépistage suppose l'utilisation d'un test efficace et adapté aux réalités locales.

**Méthodes:**

L'objectif de cette étude était de comparer les performances du frottis cervico-vaginal (FCV) conventionnel et de l'inspection visuelle à l'acide acétique (IVA) et au soluté de Lugol (IVL) couplée à la colposcopie, dans la détection des lésions du col utérin au Gabon. Des tests IVA/IVL et FCV ont été effectués chez 309 femmes gabonaises âgées de 18 à 75 ans. Des biopsies ont été réalisées en cas de résultat positif.

**Résultats:**

5 cancers épidermoïdes (1,6%) et 4 lésions précancéreuses (1,3%) ont été confirmées par l'histologie. L'IVA/IVL a présenté une meilleure sensibilité (100%) que le FCV (89%). Toutefois, le FCV est apparu plus spécifique (100% versus 92%). Avec une valeur prédictive (VP) négative de 100%, l'IVA/IVL a permis d'exclure avec certitude la présence de cancer du col lorsque le résultat était négatif, contrairement au FCV (92%). L'IVA/IVL et le FCV ont présenté des VP positives respectivement de 90% et 100%.

**Conclusion:**

Cette étude montre que l'IVA/IVL avec colposcope couplée à l'histologie en cas de résultat positif apparaît plus performante que le FCV.

## Introduction

Le cancer du col de l'utérus est la malignité du tractus génital féminin la plus fréquente dans les pays en voie de développement et demeure l'une des principales causes de mortalité dues au cancer chez la femme en Afrique Sub-saharienne [1–3]. Au Gabon, ce cancer est généralement diagnostiqué à un stade clinique avancé où l'indication radio-chirurgicale s'impose avec toutes les conséquences psychosociales et économiques qui en découlent [4]. Ainsi, entre 1984-1993, le cancer du col de l'utérus représentait 26.3% de l'ensemble des cancers féminins avec une survie à 5 ans de moins de 50% [5]. Les infections à papillomavirus humain (HPV) sont fréquentes et sexuellement transmissibles. Les souches oncogéniques, à haut risque (HR-HPV), sont les principaux facteurs de risque du cancer du col de l'utérus. La durée de l'infection par des HR-HPV a été associée au développement de lésions précancéreuses intra-épithéliales transitoires, persistantes ou évoluant en cancer [6, 7]. On évalue à une dizaine d'années la période qui sépare les premières anomalies cytologiques de l'apparition éventuelle d'un cancer infiltrant. Les vaccins préventifs, actuellement disponibles sur le marché, ciblent particulièrement les HR-HPV 16 et 18 [8]. Cependant, l'immunodépression et les infections sexuellement transmissibles potentialiseraient l'action des souches oncogènes du HPV [9]. D'autres facteurs tels qu'une parité élevée et des conditions socioéconomiques défavorisées participent également au développement des dysplasies cervicales [10]. Le cancer du col de l'utérus peut être prévenu par le dépistage et le traitement des lésions précancéreuses [11]. Une femme subissant un dépistage du cancer du col, entre 30 et 40 ans, verrait son risque de développer le cancer du col de l'utérus réduit de 25 à 36% [12]. En Occident, les programmes de dépistage des lésions précancéreuses sont essentiellement basés sur la cytologie, à savoir la réalisation du frottis cervico-vaginal (FCV). Ainsi, la morbidité et la mortalité dues au cancer du col de l'utérus dans les pays industrialisés sont en nette diminution du fait du dépistage systématique par FCV. Dans les pays en voie de développement (PVD), l'absence de programme de dépistage systématique est principalement à l'origine de la forte incidence du cancer du col de l'utérus observée dans la population féminine. En effet, la pauvreté, le faible niveau d'instruction et l'insuffisance de personnel qualifié rendent difficiles la mise en oeuvre de ce type de programmes [13, 14]. L'inspection visuelle du col de l'utérus après application d'acide acétique (IVA) et/ou de solution de Lugol (IVL) est une approche de dépistage du cancer du col de l'utérus simple, abordable et particulièrement adaptée aux réalités des PVD [15]. L'IVA/IVL présente l'avantage de dépister des lésions précancéreuses et cancéreuses opérables du col avec une sensibilité comparable et parfois meilleure à celle du FCV. Selon des études africaines, la sensibilité du test IVA/IVL varierait de 79 à 97% avec une spécificité se situant entre 87 et 96% [16, 17]. Récemment, à Libreville, plusieurs médecins spécialistes ont été formés à la technique IVA/IVL couplée à la colposcopie en vue de vulgariser le dépistage du cancer du col sur l'ensemble du territoire gabonais. Ainsi, le but de cette étude est d’évaluer les performances des tests IVA/IVL et FCV, en les comparant à l'histologie, dans la détection des lésions du col utérin au Gabon. Nous souhaitons également apprécier la maîtrise de la technique IVA/IVL par un médecin spécialiste nouvellement formé.

## Méthodes

309 femmes, âgées de 18 à 75 ans (âge moyen: 39,9 ± 10,5ans), ont été recrutées entre janvier 2013 et juillet 2014 au Centre hospitalier universitaire de Libreville (CHUL), au Centre hospitalier universitaire d'Angondjé (CHUA) et à L'institut de cancérologie de Libreville (ICL) pour une étude descriptive transversale. Plus particulièrement, cette étude visait à comparer les résultats de la colposcopie à ceux du frottis cervico vaginal (FCV). L’étude devait inclure des femmes gabonaises sexuellement actives et/ou ménopausées. Étaient exclues les femmes enceintes, les femmes présentant des saignements vaginaux et celles ayant des antécédents d'hystérectomie ou de lésions précancéreuses et cancéreuses. Après avoir été enregistrées, ces femmes devaient donner leur consentement éclairé et répondre à un formulaire comprenant des questions relatives à l’état civil et aux antécédents gynécologiques et obstétricaux. Elles étaient ensuite soumises à un FCV suivi d'une colposcopie (IVA/IVL). Le FCV a été réalisé selon la méthode conventionnelle par étalement direct sur lames des cellules recueillies à l'aide d'une spatule d'Ayre et d'une cytobrosse, puis fixé immédiatement après prélèvement. Toutes les lames ont été envoyées au laboratoire d'anatomo-cytopathologie et lues par le même pathologiste de l'ICL. La patiente était ensuite soumise à une colposcopie réalisée, par le même gynécologue, en trois étapes: un examen sans préparation, suivie d'une IVA et enfin d'une IVL. L'examen colposcopiquepermet d'observer les modifications de coloration du col et de détecter des lésions précancéreuses et cancéreuses éventuelles [18]. L'examen sans preparation a été réaliséaprès un nettoyage doux du col à l'aide de sérum physiologique. Un agrandissement optique et l'utilisation de filtres de lumière spécifiques ont facilité l'examen de la coloration du col, des micro-vaisseaux sanguins sur la surface de l'exocol et de la zone de junction squamo-cylindrique. L'IVA consiste en l'application d'acide acétique 4% sur le col de la patiente, puis en l'observation sous un fort éclairage des changements de couleur au niveau de la jonction pavimenteuse du col utérin. Les patientes IVA positif présentent une coloration blanchâtre dense, bien définie et proche de la zone de jonction ou qui touche la zone de transformation. Les patientes IVA negative ne présentent aucune coloration blanchâtre à proximité de la zone de jonction [19]. Enfin, l'IVLconsiste en l'application d'une solution iodée sur le col utérin, puis en l'observation des modifications de couleur du col. Chez les patientes IVL négatif, l'iode glycophile est normalement absorbé par l’épithélium pavimenteux du col et le colore en noir ou acajou. Les lésions précancéreuses et cancéreuses, IVL positif, n'absorbent pas l'iode car elles ne contiennent pas de glycogène, elles apparaissent alors en zones définies, épaisses, couleur safran ou jaune moutarde [19]. L'histologie est l'examen de référence pour le diagnostic des dysplasies cervicales. Aussi, des biopsies ont été réalisées au niveau des zones lésionnelles individualisées et immédiatement incluses, suivant leur prélèvement, dans du formol tamponné à 10%,puis acheminées au laboratoire d'anatomo-cytopathologie de l'ICL. Ainsi, les résultats obtenus via la colposcopie et le FCV ont pu être comparés aux resultants histologiques. Les analyses statistiques ont été réalisées à l'aide du logiciel R (version 3.1.0). Les résultats du FCV et de l'IVA/IVL couplée à la colposcopie ont été comparés. La sensibilité, la spécificité, ainsi que les valeurs prédictives positive (VPP) et négative (VPN) ont été calculées afin d’évaluer les performances du FCV, de la colposcopie sans préparation, de l'IVA, de l'IVL et de l'IVA/IVL. L'histologie a été utilisée comme test de référence.

## Résultats

### Les caractéristiques socioéconomiques et démographiques

La catégorie d’âge la plus importante est celle des 40-49 ans, 34% (Tableau 1). Ces femmes sont essentiellement célibataires (43%), relativement éduqué avec 44% des femmes déclarant avoir un niveau d’étude secondaire et 31%, un niveau d’étude universitaire. Ce sont principalement des fonctionnaires (28%), mais l'on observe aussi un taux important de femmes sans-emploi (23%). Cet échantillon populationnel apparaît majoritairement originaire du Woleu-Ntem (∼25%) et dans une moindre mesure de l'Estuaire (16%). La plupart de ces femmes sont domiciliées dans la province de l'Estuaire (67%), plus précisément à Libreville (∼17%) ainsi que dans ses banlieues nord (Akanda, 6%) et sud (Owendo, 8%).

**Tableau 1:** Caractéristiques socioéconomiques et démographiques (N = 309)

caractéristiques socioéconomiques et démographiques	Nb (%)
**Province d'origine**	
Estuaire	49 (15,8)
Haut-Ogooué	30 (9,7)
Moyen-Ogooué	16 (5,2)
Nyanga	41 (13,3)
Ogooué-Ivindo	13 (4,2)
Ogooué-Lolo	28 (9,1)
Ogooué-Maritime	16 (5,2)
Woleu-Ntem	76 (24,6)
Ngounié	40 (12,9)
**Domiciliation**	
*Estuaire*	303 (98,4)
Libreville	53 (17,2)
Owendo	25 (8,1)
Akanda	18 (5,8)
Autres	207 (67,2)
*Intérieur du pays*	5 (1,6)
**Âge**	
<20 ans	2 (0,6)
20- 29 ans	57 (18,4)
30- 39 ans	87 (28,2)
40- 49 ans	105 (34)
50- 59 ans	49 (15,9)
≥60 ans	9 (2,9)
**Catégorie socio-professionnelle**	
Fonctionnaire	86 (27,8)
Privé	57 (18,4)
Élève/Étudiant	44 (14,2)
Petits métiers	39 (12,6)
Retraités	12 (3,9)
Sans emploi	71 (23)
**Niveau d’étude**	
Primaire	66 (21,4)
Secondaire	135 (43,8)
Universitaire	94 (30,5)
Analphabète	13 (4,2)
**Statut marital**	
Célibataire	131 (42,4)
Concubinage	72 (23,3)
Mariée	93 (30,1)
Veuve	13 (4,2)

### Les facteurs de risque et autres caractéristiques gynécologiques

Plusieurs facteurs augmentent le risque de développer des lésions précancéreuses et cancéreuses du col de l'utérus (Tableau 2). Ainsi, les femmes de cette étude ont déclaré avoir eu 1 à 20 partenaires sexuels, la moyenne étant de 6,6 ± 4,1. L’âge au premier rapport sexuel était compris entre 11 et 27 ans (16,6 ± 2,4 ans en moyenne). Chez ces femmes, on a également compté 0 à 12 parités (3,4 ± 2,6 en moyenne) avec une primiparité survenue entre 13 et 34 ans (âge moyen: 19,5 ± 3,9 ans). Nous avons aussi dénombré 63% de multigestes avec au moins quatre gestités et ∼45% de femmes présentant une parité de 4 et plus, mais aussi 8% de nulligestes. D'autre part, 40.8% des patientes ont déclaré avoir déjà contracté une infection sexuellement transmissible. L'on a également noté que 48,9% des 309 patientes avaient déjà eu recours à un moyen de contraception sur des périodes non précisées: un contraceptif oral (32,36%) et/ou un préservatif (27,83%) et/ou un stérilet (1,62%). Plus particulièrement, 3,3% de ces femmes ont déclaré prendre des oestroprogestatifs au moment de l’étude. De plus, 2,6% des femmes recrutées sont tabagiques (et 32% déclarent consommer de l'alcool), la fréquence de la consommation n’étant pas précisée. Finalement, seulement 15,2% des femmes recrutées avaient subi au préalable un frottis cervico-vaginal, la majorité des patientes n'ayant jamais été testée (84.5%).

**Tableau 2:** Facteurs de risque

	Moyenne±d.s./ Prévalence (%)	Nombre
*Caractéristiques reproductives*		
Âge (années)	39,9 ± 10,5	309
Âge au 1er rapport sexuel (années)	16,6 ± 2,4	307
Nombre de partenaires sexuels	6,6 ± 4,1	309
Parité	3,4 ± 2,6	309
Âge à la Primiparité (années)	19,5 ± 3,9	273
Âge de la Ménopause (années)	48,9 ± 4,9	59
*IST*		
HIV	8,77	59
Chlamydia	35,92	309
Syphilis	1,62	309
Gonococcie	4,85	309
Autres IST	2,59	309
*Tabac*	2,60	309
Oestroprogestatifs*	32,36	309

IST: 35,9% des femmes ont déclaré des antécédents d'infection à Chlamydia trachomatis et/ou à la syphilis (1,62%) et/ou à la gonococcie (4,85%) et/ou à un autre type d'IST (2,59%). Sur les 309 patientes, seules 59 connaissaient leur statut sérologique vis-à-vis du virus d'immunodéficience humaine (VIH), parmi lesquelles 5 ont déclaré être séropositives.

* 32,4% des femmes déclarent avoir déjà utilisé des oestroprogestatifs sur des laps de temps non-précisés et 3,3% déclarent en consommer au moment de l’étude; d.s.=déviation standard

### Résultats des tests de dépistage

L'Inspection visuelle à l'acide acétique et au soluté de Lugol couplée à la colposcopie. Une IVA/IVL effectuée à l'aide d'un colposcope a été réalisée sur les 309 patientes (Tableau 3). La colposcopie sans préparation (avant l'ajout d'acide acétique ou de Lugol) a permis de repérer 121 cols d'aspect anormal (rouge, 39,2%) incluant 7 lésions suspectes et 188 cols d'aspect normal (rose, 60,8%). Parmi ces derniers, on a observé des modifications et lésions non-dysplasiques du col utérin à savoir 4 kystes de Naboth, 18 ectropions et 99 inflammations. Par l'IVA, 26 (8,4%) cols acidophiles ont été identifiés, parmi lesquels 18 (5,8%) transformations atypiques de grade 1 (TAG1) et 8 (2,6%) transformations atypiques de grade 2 (TAG2). Par l'IVL, 116 (37,5%) cols iodo-négatifs ont été détectés, incluant 98 (31,7%) cols avec un aspect de colpite (anomalie bénigne). En somme, l'IVA/IVL a permis de détecter 194 (62,8%) cols normaux et 104 (33,7%) cols inflammatoires. Parmi les 4 cols normaux biopsiés, 3 (75%) ont été confirmés par l'histologie (tableau 4). 3 (50%) des 6 cols inflammatoires biopsiés ont été confirmées par l'histologie, un 4ème s'est révélé être une lésion CIN1. 7 (1,9%) lésions précancéreuses ont aussi été identifiées par l'IVA/IVL dont 4 (57%) ont été confirmées par l'histologie, une 5ème s'est avérée être un cancer épidermoïde. Finalement, les 4 (1,3%) lésions cancéreuses détectées par l'IVA/IVL ont été confirmées (100%) par l'histologie. De plus, une patiente (0,3%) a présenté un col qu'il n'a pas été possible de classifier (aspect indéterminé).

**Tableau 3:** Résultats de la colposcopie et de la cytologie (N = 309)

Tests	Résultats	Nb de cas (%)
**Colposcopie**		
***Sans préparation***		
	Négatif	188 (60.84%)
	Positif	121 (39.16%)
***IVA***		
	Négatif	283 (91,58%)
	TAG1	18 (5.82)
	TAG2	8 (2.59)
***IVL***		
	Négatif	193 (62.46%)
	Colpite	98 (31.71%)
	Positif	18 (5.82%)
***IVA/IVL***		
	Normal	194 (62,78%)
	Inflammatoire	103 (33,33%)
	Lésion Précancéreuse	7 (2,27%)
	Lésion Cancéreuse	4 (1,29%)
	Indéterminé	1 (0,32%)
**Cytologie**		
	Normal	185 (59.87%)
	Inflammatoire	109 (35.27%)
	ASC-US	2 (0.65%)
	HSIL	4 (1.29%)
	Lésion Cancéreuse	4 (1.29%)
	Indéterminé	5 (1.62%)

Dans les résultats de l'IVA, TAG1 désigne une transformation atypique de grade 1 et TAG2 une transformation atypique de grade 2 faisant référence aux lésions précancéreuses et cancéreuses. Dans les résultats cytologiques (FCV), ASC-US (atypical squamous cell of undetermined significance) désigne des atypies de cellules malpighiennes de signification indéterminée. HSIL (High grade squamous intraepithelial lesions) désigne les lésions malpighiennes intra-épithéliales de haut-grade.

**Tableau 4:** Résultats de l'Histologie

Test	Résultats	Cancer	CIN 2/3	CIN1	Inflammatoire	Normal	Total
**IVA/IVL**	Cancer	4 (80%)	0	0	0	0	4
	Lésion précancéreuse	1	4(100%)	0	1	1	7
	Inflammatoire	0	0	1	3 (60%)	2	6
	Normal	0	0	0	1	3 (50%)	4
**FCV**	Cancer	4 (80%)	0	0	0	0	4
	HSIL	1	3 (75%)	0	0	0	4
	ASC-US	0	1	0	0	0	1
	Inflammatoire	0	0	1	4 (80%)	2	7
	Normal	0	0	0	1	4 (67%)	5
	**Total**	**5**	**4**	**1**	**5**	**6**	**21**

CIN1 = néoplasie cervicale intra-épithéliale de bas grade (grade 1); CIN2 et 3 = néoplasie cervicale intra-épithéliale de haut grade (grade 2 et grade 3, respectivement).

### La cytologie

**Figure 1:**
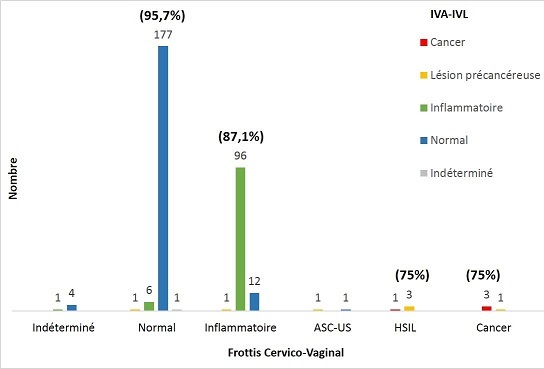
Comparaison entre les résultats des tests IVA/IVL et FCV (N = 309). 177 (95,7%) des 185 cols normaux par le FCV, ont été confirmés par l'IVA/IVL comme 96 (87,1%) des 109 cols inflammatoires. 3 (75%) des 4 lésions de haut grade (HSIL) ou des cancers identifiées par le FCV ont été classées comme lésions précancéreuses ou cancers par l'IVA/IVL.

Les résultats des 309 frottis effectués sont rapportés selon la classification de Bethesda [20] (Tableau 3). Ainsi, 109 frottis (35,3%) ont identifiés des cols inflammatoires, 5 frottis (1,6%) étaient classés non contributifs, alors que 185 frottis (59,9%) sont apparus normaux. 4 des 5 (80%) cols normaux biopsiés ont été confirmés par l'histologie (Tableau 4). Parmi les 2 frottis (6,6%) classés ASC-US par la cytologie, l'un s'est révélé être une lésion CIN2 selon l'histologie. Finalement, le FCV a détecté 4 (1,3%) lésions de haut grade (HSIL) dont 3 (75%) ont été confirmées par l'histologie (CIN3), la quatrième s'est avérée être un cancer épidermoïde. Finalement, les 4 (1,3%) cancers épidermoïdes détectés par le FCV ont été confirmés (100%) par l'histologie. La comparaison de l'IVA/IVL et du FCV a montré que concernant la détection des cols normaux, inflammatoires, et des lésions précancéreuses et cancéreuses, les résultats corrèlent respectivement à 96%, 87%, et 75% (Figure 1). 31% de cols inflammatoires ont été détectés par les 2 tests à la fois, chez les 309 patientes.

### L'histologie

Un total de 21 biopsies ont été réalisées chez toutes les patientes chez lesquelles des lésions précancéreuses, de haut grade ou un cancer du col de l'utérus étaient suspectés. De plus, quelques patientes à priori non-affectéeset sélectionnées aléatoirement ont également été testées (Tableau 4). Parmi les 309 femmes analysées, cinq cas de cancer épidermoïde (1,6%) ont finalement été définitivement confirmés par l'examen histologique, soit 23,8% des 21 biopsies réalisées. Ces femmes étaient âgées de 27 à 70 ans (50,4 ± 15,6 ans en moyenne). Chez les 309 femmes testées, quatre lésions précancéreuses (1.3%) ont été confirmées par l'histologie, une lésion CIN1 et 3 lésions CIN3 représentant 19% des 21 biopsies effectuées. Les femmes présentant des lésions précancéreuses avaient entre 28 et 55 ans (46±14,5 ans en moyenne). Par ailleurs, l'histologie a aussi détecté des cols normaux et inflammatoires, soit respectivement 28,2% et 23,8% des 21 biopsies réalisées. L'IVA/IVL a pu identifier comme lésions précancéreuses les 4 cas de lésions CIN2/3 confirmés par la biopsie, alors que le FCV n'en a identifié que 3 (75%) comme lésions de haut-grade. Les 2 tests ont pu détecter 4 (80%) des 5 cancers épidermoïdes confirmés par la biopsie.

### Spécificité et sensibilité

Nos résultats montrent que l'IVA/IVL couplée à la colposcopie apparaît comme une méthode de dépistage performante (Tableau 5). En effet, elle présente une spécificité de 92% et une sensibilité de 100%. Cet examen a également une excellente VPN (100%) permettant d'exclure avec confiance la présence de cancer du col si le résultat est négatif, contrairement à la cytologie (∼92%). Toutefois, un résultat positif d'IVA/IVL couplée à la colposcopie n'indique pas formellement la présence de cancer du col de l'utérus (VPP de 90%). L'approche consistant à soumettre systématiquement les patientes présentant un résultat positif à un examen histologique devrait faciliter la mise à l’écart des résultats faux positifs de façon effective.

**Tableau 5:** Sensibilité et spécificité de la colposcopie et de la cytologie, en fonction de l'histologie (N = 21)

	Tests	Résultat	Histologie Négative	Histologie Positive	Sensibilité (IC 95%)	Spécificité (IC 95%)
**Colposcopie**	Sans préparation§	Négative	4	0	100% (66.21-100.00)	33.33% (10.13-65.05)
		Positive	8	9		
	IVA*	Négative	12	1	88.89% (51.74-98.16)	100.00% (73.35-100.00)
		Positive	0	8		
	IVL**	Négative	10	0	100.00% (66.21-100.00)	83.33% (51.58-97.42)
		Positive	2	9		
	IVA/IVLβ	Négative	11	0	100.00% (66.21-100.00)	91.67% (61.46-98.61)
		Positive	1	9		
**Cytologieα**		Négative	12	1	88.89% (51.74-98.16)	100.00% (73.35-100.00)
		Positive	0	8		

§ Valeur prédictive positive (VPP): 52.94% (95% IC: 27.86% - 76.96%), valeur prédictive négative (VPN): 100.00% (95% IC: 40.23% - 100.00%).

* VPP: 100.00% (95% IC: 62.91% - 100.00%), VPN: 92.31% (95% IC: 63.90% - 98.72%).

** VPP: 81.82% (95% IC: 48.24% - 97.18%), VPN: 100.00% (95% IC: 68.97% - 100.00%).

β VPP: 90.00% (95% IC: 55.46% - 98.34%), VPN: 100.00% (95% IC: 71.33% - 100.00%).

α VPP: 100.00% (95% IC: 62.91% - 100.00%), VPN: 92.31% (95% IC: 63.90% - 98.72%). IC 95% = Intervalle de Confiance à 95%. Pour l'IVA, TAG2 était considéré positif. Pour l'IVL, les iodo-négatifs sans colpite étaient considérés positifs. Pour l'IVA/IVL, les lésions précancéreuses et les cancers étaient considérés positifs. Pour la cytologie (FCV), les lésions de haut-grade (HSIL)et les cancers étaient considérés positifs. Pour l'histologie (biopsie), les lésions CIN2 et CIN3 et les cancers étaient considérés positifs.

## Discussion

Le dépistage systématique des lésions précancéreuses et cancéreuses du col de l'utérus n'est pas organisé au Gabon. La mise en place de modalités organisationnelles implique l'utilisation d'un test efficace et adapté auxréalités gabonaises. Cette étude est une évaluation comparative du FCV et de l'IVA/IVL couplée à la colposcopie, dans la détection de lésions cancéreuse et précancéreuse chez les femmes gabonaises. Les informations recueillies nous ont également permis de dresser un profil socioéconomique des femmes ayant pris part volontairement à cette étude. Nous avons détecté 5 cancers épidermoïdes (1,6%) et 4 lésions précancéreuses (1,3%) chez les 309 femmes testées. Seules 15.2% de ces femmes avaient préalablement subi un FCV. En effet, l'absence de programme de dépistage systématique explique le faible taux de diagnostic précoce de lésions précancéreuses et cancéreuses du col de l'utérus obtenu dans la plupart des PVD. Au Gabon, il n'existe que 3 anatomopathologistes exerçant dans la région de Libreville ce qui complique la mise en ouvre de dépistage systématique utilisant le FCV, à l’échelle nationale. Nos résultats montrent que les tests FCV et IVA/IVL avec colposcope sont fortement corrélés (87% et plus) en ce qui concerne la détection des cols normaux et non-dysplasiques. Quatrecas de lésions CIN2/3 ont été confirmés par l'histologie et l'IVA/IVL les identifie toutes comme lésions précancéreuses, alors que le FCV n'en identifie que 3 (75%) comme lésions de haut-grade. Les 2 tests ont détecté 4 (80%) des 5 cancers épidermoïdes confirmés par l'histologie, le 5^ème^ ayant été classé lésion précancéreuse ou de haut grade. Plus particulièrement, l'IVA/IVL présente une spécificité de 92% et une sensibilité de 100%. Ce test devrait permettre d’éliminer avec confiance la présence de cancer du col lorsque le résultat est négatif (VPN de 100%). De plus, si l'IVA/IVL présente une VPP de 90%, l'application systématique d'un examen histologique à tous les résultats positifs (lésions précancéreuse et cancéreuse) devrait faciliter l'exclusion efficace de tous les faux positifs.

L'ensemble de la population féminine évaluée dans cette étude est composée de femmes âgées de 18 à 75 ans (âge moyen 39,9 ± 10,5 ans) et relativement instruites, la plupart étant des fonctionnaires (27.8%). Il n'est plus à démontrer que l'activité sexuelle constitue le principal facteur de risque pour le développement du cancer du col de l'utérus. L’âge au premier rapport sexuel de l'ensemble des femmes recrutées variait entre 11 et 27 ans (16,6 ± 2,4 ans en moyenne)pour un nombre de partenaires sexuels moyen de 6,6 ± 4,1 et une utilisation restreinte de moyens contraceptifs. Une activité sexuelle non protégée a été associée à une susceptibilité accrue à contracter des infections sexuellement transmissibles (IST) dont l'action sur l'inflammation du col et l'immunité locale va favoriser le développement de dysplasies cervicales [21]. Ainsi, 40.8% des patientes ont déclaré avoir déjà contracté une IST, les plus fréquentes étant les infections à *C. trachomatis* (35.9%) et *N. gonorrhoeae* (4,8%). Ces dernières ont été reliées au développement de cervicites chez des femmes africaines [22, 23]. Aussi, dans cette étude, il apparaît que l'inflammation du col de l'utérus est la lésion non-dysplasique prépondérante chez la femme gabonaise, avec plus de 30% de cas détectés à la fois par l'IVA/IVL et le FCV. L'on note que le FCV communément réalisé au Gabon est un frottis conventionnel sur lames, la technologie pour le frottis en milieu liquide n’étant pas disponible. Le contexte très inflammatoire, souvent observé dans notre population féminine, peut rendre la lecture du FCV conventionnel delicate et conduire à des interprétations erronées [24]. Aussi, un frottis de contrôle est recommandé après le traitement de l'inflammation. D'autre part, le délai important d'attente des résultats et/ou les coûts associésau FCV découragent certaines femmes quant à la prise des résultats [12, 14]. À l'opposé, l'IVA/IVL est un test peu coûteux avec une obtention des résultats immédiate, lors de la première visite. Plusieurs facteurs pourraient affecter la précision des résultats présentés dans cette étude. Les prévalences des IST, incluant notamment le VIH, ont été calculées sur la base des déclarations des femmes constituant la cohort et sont de ce fait très certainement largement sous-estimées. Plus particulièrement, les prévalences du virus de l'herpès simplex 2 ou des souches oncogènes de HPV sont inconnues dans cette cohorte. De plus, la taille de la cohorte est restreinte du fait de diverses contraintes financières et techniques. D'autre part, l'on note que la spécificité et sensibilité de l'inspection visuelle peuvent varier selon l'expérience du professionnel effectuant l'examen, la source de lumière utilisée ou la préparation des solutions employées et leurs conditions de stockage [25]. Toutefois, dans la présente étude, l'IVA/IVL a été effectuée par un seul gynécologue qui a pu accéder, en peu de temps, à un bon niveau d'expertise technique.

## Conclusion

En définitive, nos résultats montrent que l'IVA/IVL avec colposcope couplée à l'histologie en cas de suspicion de lésions précancéreuses et cancéreuses est plus performante que le FCV dans notre population. De plus, la simplicité de la méthode, la rapidité de l'obtention des résultats et les coûts peu élevés associés font que ce test est particulièrement adapté au contexte socioéconomique gabonais.
